# Supplementary material for: Suppression of interdiffusion-induced voiding in oxidation of copper nanowires with twin-modified surface
Source: Nat Commun. 2018 Jan 23;9:340. doi: 10.1038/s41467-017-02154-3 (PMC5780419; doi:10.1038/s41467-017-02154-3)
Supplement: Supplementary file 1 — Supplementary Information [file 41467_2017_2154_MOESM1_ESM.pdf]

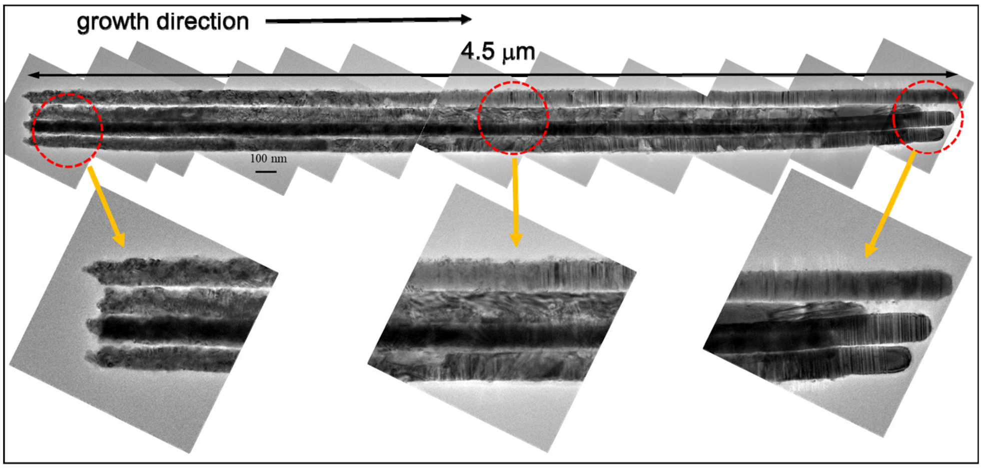

Supplementary Figure 1 Bright-field TEM images of bundled CuNWs released from an AAO template. The CuNWs exhibit nanocrystalline microstructure in the initial growth stage (left) and transform into nanotwinned structure with dense CTBs perpendicular to the growth direction in the latter stage (right).

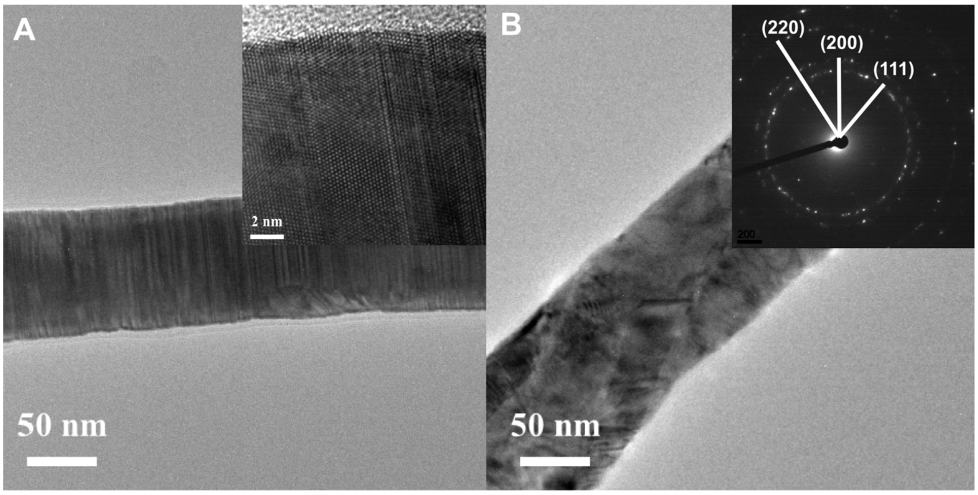

Supplementary Figure 2 Bright-field TEM images of the CuNWs with (A) nanotwinned and (B) nanocrystalline microstructures. The insets are the HRTEM image of the nanotwinned CuNW and the selective area electron diffraction pattern of the nanocrystalline CuNW.

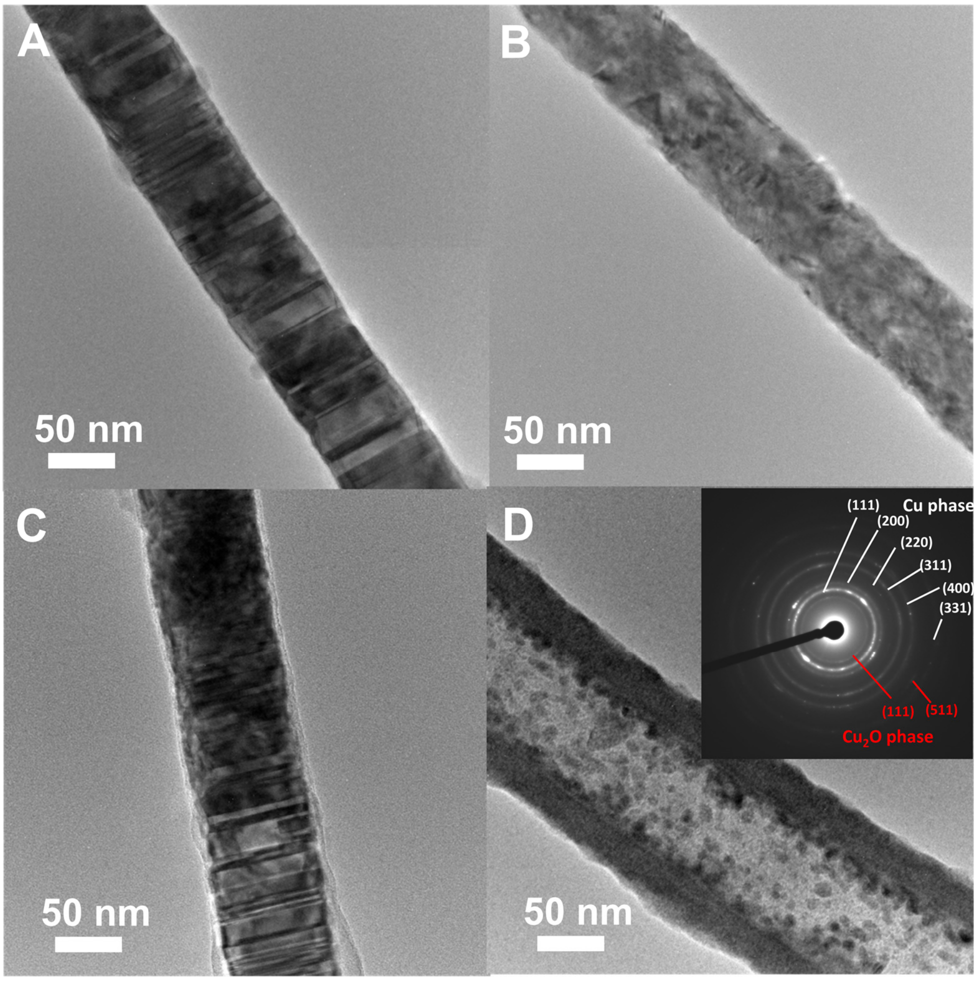

Supplementary Figure 3 Bright-field TEM images of the nanotwinned and nanocrystalline CuNWs. (A, B) before thermal treatment; (C, D) after heating at 150 °C in air for 1 h. The inset of (D) is the selective area electron diffraction pattern of the nanocrystalline CuNW with oxide shell.
